# The Perceptual Organisation of Visual Elements: Lines

**DOI:** 10.3390/brainsci11121585

**Published:** 2021-11-30

**Authors:** Liliana Albertazzi, Luisa Canal, Rocco Micciolo, Iacopo Hachen

**Affiliations:** 1 LabExP, Department of Humanities, University of Trento, 38122 Trento, Italy; 2 Department of Psychology and Cognitive Sciences, University of Trento, 38122 Trento, Italy; luisa.canal@unitn.it (L.C.); rocco.micciolo@unitn.it (R.M.); 3 Neuroscience Area, International School for Advanced Studies (SISSA), 34136 Trieste, Italy; iacopo.hachen@alumni.unitn.it

**Keywords:** lines, spatial elements, visual geometry, visual lines, visual space

## Abstract

The aim of this study is to verify the conditions under which a series of visual stimuli (line segments) will be subjectively perceived as visual lines or surfaces employing four experiments. Two experiments were conducted with the method of subjective evaluation of the line segments, and the other two with the Osgood semantic differential. We analysed five variables (thickness, type, orientation, and colour) potentially responsible for the lines’ categorisation. The four experiments gave similar results: higher importance of the variables thickness and type; general lower significance of the variable colour; and general insignificance of the variable orientation. Interestingly, for the variable type, straight lines are evaluated as surfaces more frequently than curved lines and perceived as geometrical, flat, hard, static, rough, sharp, bound, sour, frigid, masculine, cold and passive. Curved lines are prevalently evaluated as lines, and categorised as organic, rounded, soft, dynamic, fluffy, blunt, free, sweet, sensual, feminine, warm and active. These results highlight the specificity of perceptual characteristics for the considered variables and confirm the relevance of the characteristics of variables such as thickness and type.

## 1. Introduction

### 1.1. Visual Lines

This paper shows that the understanding of visual line generally adopted by visual science requires reconsideration. In visual space there are not Euclidean lines, but so to say lineoids, i.e., something appearing similar to lines [1]. Euclidean lines do not exist in nature as well, where we have presentations of strands of grass or of skinny leaves such as those of the dasylirion or the thin branches or the tendrils of the olive trees, whose dimensions fluctuate among lines, surfaces and volumetric appearance.

Visual lines may have different appearances and behave differently in perceptual space. They can appear simply as lines of their own on a background, or otherwise: boundaries between regions or surfaces [2,3,4,5,6]; line drawings [7,8,9,10,11,12]; edges (surface discontinuities) [10,13]; contours of flat figures (silhouettes) [10]; factors of visual organisation as in cases of contour rivalry [7,13]; generators and/or shape deformers (as in the Rubin vase [5]; and the Hering illusion [14]); inducers of phenomenal transparencies [15]; margins of forms in case of brightness contrasts with the adjacent areas [16]; gradients of depth [17]; textural elements (hatch line) [10]; marks or strokes (blobs of paint on canvas or paper), cracks or ruptures on surfaces [8,10], and so on. In some cases, such as marks, strokes, and cracks in surfaces, lines are very close in appearance to surfaces [18]. Hence, the structural ambiguity of the definition of visual lines.

Each of these appearances plays a specific role in visual organisation. To obtain a scientific phenomenology of lines, their behaviour must be identified and analysed as such, although often they may appear in the same configuration (for example, lines as such, edge lines, and texture lines in Steinberg’s drawings [10] (p. 111). Lines’ appearances are also characterised by expressive and connotative properties, appearing lively or inanimate, rough or smooth, hard or weak, and so on. Sharp lines, for example, are associated with aggression [19]. These attributes of visual forms, besides Gestalt psychology [7,20], have also been significantly addressed by the realm of art (consider how they appear in Klee’s paintings, Landscape physiognomic, 1931, and Temperaments, 1929). Artists devoted much effort to defining lines behaviour, until making it their daily obsession, as with Klee. In visual arts, indeed, we have most of the drawn phenomenology of visual lines, able to contribute to their classification [21].

Lines in pictorial space can be used to identify boundaries of areas of different colours or tones on a surface [8], to represent transparent planes [15], or the recognition of smooth objects following consistent ‘cognitive’ rules [22], etc. The variety of the great number of ways of appearance of lines cast doubt on their univocal representation in terms of axiomatic geometries. They can be represented in Euclidean geometry, but in so doing they lose most of their basic perceptual characteristics, such as colour and expressivity.

Visual lines can also be represented in drawings, painting, and graphics where instead they keep some of their basic, inherent, qualitative, non-metrical characteristics [10,23,24,25].

### 1.2. Phenomenology of Visual Lines

Phenomenologically, a visual line must have some minimal thickness to be visible, and a thick line behaves differently from a thin line; it may be one- or bi-dimensional (consider a hyper-compressed line reduced to its minimum, or imperceptibly circular or even oval); it is localised in visual space; it is coloured; it possesses figural characteristics such as parts potentially overlapping (think of intermediate unmarked points dividing a broken line), and trait characteristics (thick, curved, zigzagged, blurred). The phenomenology of lines is broad, for example there are smooth boundaries of a zigzagged line or zigzagged boundaries of a curved line, and so on.

Thus, for a line to be visual, there must be at least an organisation of type (straight, curved), thickness, colour, and orientation. Visual lines, similar to visual points, might also have expressive characteristics (appearing good or bad, stable or unstable, harmful or harmless), relatively to their thickness, orientation, and degrees of freedom in a configuration [25]. Lines can be amodal, continuing behind an occluder, and can possess multi-directional attributes.

To understand the nature of visual lines and identify the boundaries of the category, after observing and describing the multiple forms of their appearance, the dimensional components of lines should be experimentally verified. The analysis to be conducted closely resembles that of Katz’ on colour categorisation [26]. Only when the ‘grammar of seeing’ lines is established [11,27], one is entitled to look for their definition and classification as elements of a visual geometry, and their behaviour in visual space.

In our study, we started to explore some of the conditions for the subjective identification of lines in the visual field, the characteristics that render them distinguishable from visual surfaces (i.e., parts of space), and their expressive characteristics. The aim was to identify visual lines through their empirically tested characteristics, based on subjective judgments in first-person account.

Our study was limited to the characteristics of the line as such. The study does not consider lines as contours, junctions, edges, or even line drawings as mainly addressed by vision and computer science [28,29,30,31,32]; similarly, we do not address the issue of their neural substrate of such features of visual representation of natural or artificial objects in the visual cortex (starting from the pioneering studies of Hubel & Wiesel [33,34]).

The study has two systematic backgrounds. The first one is experimental phenomenology [27,35,36,37,38]. To conceive a geometry adequate for visual appearances, instead of starting from axiomatic geometry and its definitions to analyse the properties of visual lines, the study analyses and experimentally explores their characteristics as subjectively perceived. The same would hold for the analysis of visual points, surfaces and volumes. Axiomatic geometry, in fact, provides an idealization (in lines, points, length, etc.) of physical properties of objects that it is supposed to represent, while perceiving in awareness neither refers to nor evaluates physical magnitudes. Furthermore, in perception the discriminative thresholds are statistical in nature, therefore the same stimulus can cause the corresponding vision or not.

Language is obviously an obstacle, because of its widespread synonymy also in science, which leads to using the same terms (e.g., line) for physical segments, idealized and/or definitional entities, perceptual appearances, and so on [39].

The second systematic ground comes from the theory and the works of the visual arts. Kandinsky, in particular, was a source of inspiration for the line segments used in the experiments (see below, how they were produced). In this respect, the overall guiding idea of the study is the close relationship between the Gestalt laws of seeing and their representation in the works of art.

## 2. Materials and Methods

### 2.1. The Study

We addressed the nature of visual lines according to the following questions: when, in *seeing*, does a line cease to appear as a line and when does it start to appear as a stroke, or a surface, even if small in extent? Does the type, thickness, orientation and colour (chromatic and achromatic) of the line and of its background have an influence on its categorisation? And to what extent? Do any of these dimensions have the same weight for straight and curved lines to be categorised and named as such? And, from a general viewpoint, is it possible to identify the psychometrics of these entities, reflecting their appearance?

To answer these questions, in a series of four experiments, we draw a series of (physical) line segments, both straight and curved, to test the influence in their categorisation of the following dimensions: thickness, type (if they are visually straight or curved), colour/background, and orientation. We hypothesised that in the case of positive results, these might also help to shed light on the requirements of a geometry of phenomenal visual space.

### 2.2. Participants

All participants were recruited by e-mail from students in the Department of Engineering, and in the Department of Humanities at the University of Trento, Italy. The address list of the students was provided by the student office. The experiments were also included in the training activities of the undergraduates attending the Thesis Laboratory (LaTe) of the Department of Engineering. Participation in the entire cycle was considered equivalent to one of the presentations of the progress of the thesis work. The experiments were also included in the training and professional activities replacing the internship for the students of the three-year degree and the specialization of the Department of Humanities. All the subjects signed an informed consent form. The information collected on the form concerned nationality (all the subjects were Italian), visual acuity (vision corrected by glasses, with lenses, and normal vision), normal colour vision (tested by Ishihara test), familiarity with colour acquired in work, artistic and educational settings. In the experiments, the only exclusion criterion was defective colour vision. None of the participants had education in visual arts; some of them had musical education. The experiments reported here complied with the ethical guidelines of the University of Trento.

No time restrictions were imposed, the participants could observe the line segments with ease, although they were instructed not to refer to past experience or domain of expertise (at least consciously) in their evaluations (this advice was particularly relevant for students of the Engineering Department). Participants were seated at a desk. The distance from the centre of the screen to the eye was about 65 cm and the presentation was performed in binocular vision. Chin supports were not used, but during each session the postures of the participants were checked and corrected if their chests approached the screen, or their backs were hunched.

### 2.3. Stimuli and Apparatus

The four experiments were carried on in the Experimental Phenomenology Laboratory (LabExP) at the Department of Humanities, Trento University.

The laboratory had constant and controlled lighting conditions (ca. 10 lx on the average in the room, given by a halogen lamp). The colours were produced on a monitor Eizo Color edge mod. CG276 (68 × 27 cm), P7N OFTD1846 75Q, Mfd. 2013.05.24, S/N 23816053 A (resolution 2560 × 1440). The software used to calibrate the monitor was Eizo Corporation, Color Navigator 6 v.6.4.0.5. The calibration guaranteed a white D65, a 2.2 gamma, 120 cd/m^2^ luminosity, maximum contrast. The monitor was recalibrated at the beginning of each session.

The stimuli were *physical* line segments (N.B. from here onwards: “line segments”), characterised by the following variables:

Type: straight and curved line segments. Both types were chosen to cover broadly the same amount of space between the two extremities in order to verify whether they might appear of different length and consequently influence the evaluation (i.e., factually, curved line segments were metrically longer than the straight ones).

Thickness: of five different degrees (mm: 0.5, 1.25, 3.1, 7.8, 19.5 of average thickness), in order to verify if and eventually which was the visible boundary between lines and surfaces.

Orientation: horizontal, vertical, and two diagonals (45 degrees). Following Kandinsky’s theory [29] (pp. 121–130), we labelled the diagonals as “harmonic” (lower left—top right) and “disharmonious” (lower right—top left).

**Figure 1:**
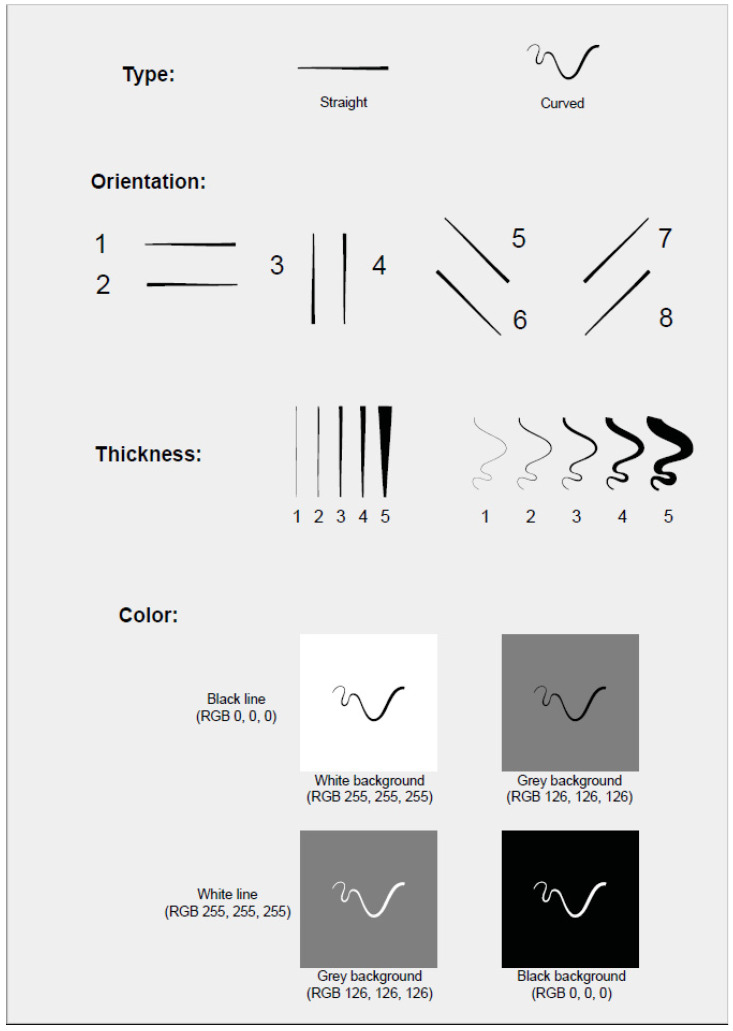
Type, orientation, thickness, background/colour of lines (1st and 3rd experiments).

**Figure 2:**
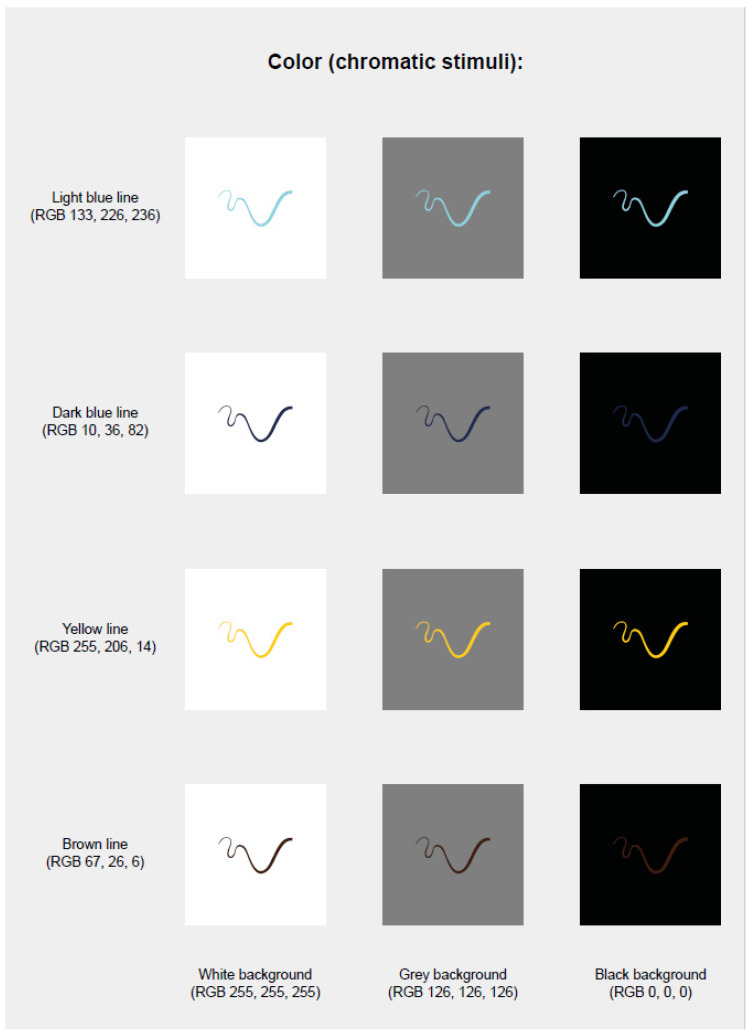
Background and colour of lines (2nd and 4th experiments).

Colour: black (RGB 0, 0, 0), white (RGB 255, 255, 255) (1st and 3rd experiments; Figure 1); light blue (RGB 133, 226, 236), dark blue (RGB 10, 36, 82), brown (RGB 67, 26, 6), yellow (RGB 255, 206, 14) (2nd and 4th experiments; Figure 2). We chose blue and brown isoluminant from a perceptual viewpoint (although not from a colourimetric viewpoint, having different L *, although only slightly). Isoluminant colours were obtained in the following way: we presented on the monitor screen a series of 30 adjacent lines 10” thickness and 8° 30′ vertical length, of alternate colours, to be observed at a distance of about 75 cm at the same adaptation state as during the experiment. One colour was fixed and the second could be varied in power until the border between the two colours was the minimally distinct, i.e., they were perceptually isoluminant [40].

Background: black, white, grey (for all the experiments).

The line segments were not produced following a mathematical function, but through the following steps. First, line segments were hand-drafted by a professional graphic designer with different techniques (pen, marker, or calligraphy nib), so that thickness variations would arise naturally by variations in hand pressure. We reasoned that hand drawings would constitute more natural stimuli, therefore closer to perceptual prototypes. We specifically sought to mimic Kandinsky’s style as observed in his work *Point and Line to Plane* (orig. 1926), where the author’s drawings are meant to illustrate perceptual primitives and categories. In a second phase, to have better control over the details and to avoid confounds that might arise by drawing each stimulus individually, we created new line segments in a vector-based software by using the manual sketches as a template. Specifically, we obtained two vectorial line templates (‘straight’ and ‘curved’) mimicking the trait and the line ends observed on paper. From each template, we obtained five different stimuli differing in their average thickness, starting from a logarithmic scale of five thicknesses. We further adjusted the average thickness of each stimulus until they appeared equally different from one another. Details were curated by prioritizing appearances instead of metric features, i.e., perceptual continuity between different thickness levels, as well as similarity to natural hand drawings. The resulting line segments (average thickness: 0.5 mm, 1.25 mm, 3.1 mm, 7.8 mm, 19.5 mm) were subject to test in a pilot experiment, to evaluate their effectiveness and specially to ensure uniformity in the perceptual transition between ‘line’ and ‘surface’. Minor adjustments to the local width of specific line segment parts were performed after the pilot results. Due to the above steps, straight segment lines involve a (mostly) monotonic increase in width, as if they were drawn in a single trait. Instead, curved segment lines involve multiple traits, corresponding to pressure and thickness variations. Indeed, curved segment lines are more complex perceptual objects, in which the observer can identify multiple salient parts.

The measures of the final line segments were the following:

Straight line segments: Thickness 1 (Max: 0.4 mm; Min: 0.2 mm); Thickness 2 (Max: 1.1 mm; Min: 0.5 mm): Thickness 3 (Max: 3 mm; Min: 1 mm); Thickness 4 (Max: 4.6 mm; Min: 1.2 mm); Thickness 5 (Max: 11.5; Min: 2.2 mm) Length: 75 mm

Curved line segments: Thickness 1 (Max: 0.2 mm; Min: 0.02 mm); Width of the rectangle bounding the stimulus (vertical orientation): 28 mm; Height of the rectangle bounding the stimulus (vertical orientation): 59 mm; Thickness 2 (Max: 0.9 mm; Min: 0.14 mm); Thickness 3 (Max: 2.2 mm; Min: 0.35 mm); Thickness 4 (Max: 5.9 mm; Min: 0.9 mm);Thickness 5 (Max: ca 12 mm; Min: 1.9 mm); Width of the rectangle bounding the stimulus (vertical orientation): 38.5 mm; Height of the rectangle bounding the stimulus (vertical orientation): 61.5 mm.

To ensure the replicability of the experiments, all stimuli used are included in the Supplementary Materials.

### 2.4. Tasks and Procedure for the Series of Experiments

In two tasks (Experiments 1 and 2) the participants were asked to evaluate whether the line segments presented on the screen were visual lines or surfaces. In another two tasks (Experiments 3 and 4), conducted with a modified version of the Osgood semantic differential, the participants were asked to evaluate the line segments according to the pairs of contraries presented on the screen. Precisely:

In Experiment 1, the achromatic (white, black) line segments were randomly presented according to the dimensions of thickness, type, orientation, and background.

In Experiment 2, the same line segments were coloured in four different hues (light blue, dark blue, brown, yellow) and randomly presented according to the dimensions of thickness, type, orientation, and background.

In Experiment 3, the same achromatic line segments of Experiment 1 were evaluated on a list of pairs of contraries, and randomly presented according to the dimensions of thickness, type, orientation, and background.

In Experiment 4, the same-coloured line segments of Experiment 2 were evaluated on a partially different list of pairs of contraries (including dimensions calibrated on connotative properties of colour, such as warm/cold or expansion/contraction), and randomly presented according to the dimensions of thickness, type, orientation, and background. The choice of the pairs of contraries in the two lists was derived partly by Kandinsky’s study and observations on the appearance of lines in pictorial space [24], and partly from our main hypothesis—tested in previous works [41,42]—of the inherent cross-modality and expressiveness of shapes in visual space, as illustrated in works of art. The list of pairs of contraries in Experiment 4 was partially different from the list of Experiment 3, presenting colour related adjectives.

#### 2.4.1. Experiment 1

**Stimuli**

The stimuli, one straight and one curved line segments with four different orientations and five levels of thicknesses, were presented in two achromatic colours (white, black) on three achromatic backgrounds (white, black, and grey) for a total of four combinations (white on black background, white on grey background, black on white background, black on grey background). Therefore, a total of 160 line segments (presented in random order) were considered.

**Task and Procedure**

In this case, 30 participants were presented the line segments appearing on the screen in random order. The participants were asked to evaluate whether the 160 line segments were visual lines or surfaces.

The participants were given the following instructions:

The experiment consists of the subjective evaluation of line segments that appear on the screen. The line segments will be randomly presented in black and white on black, white, and grey backgrounds respectively. The participant must express her subjective perception whether the line segments are lines or surfaces. There are no wrong answers, the evaluation is subjective. However, please pay close attention to the task. Participants have as much time as desired to perform the assessment task, but must try not to respond based on past experience.

**Statistical Methods**

A logistic regression model including the random effects of participants was employed to evaluate the raw effect of each variable on the probability of classifying a line as a ‘surface’. A multivariate logistic regression model including the random effects of participants was employed to evaluate the joint effect of the considered variables on the probability of classifying a line as ‘surface’. Analyses were performed using STATA 13 [43].

**Results**

Table 1 show the number of choices (line/surface) made by the 30 subjects according to the variables considered. A significant effect was found for all these variables but orientation.

As expected, the most striking effect was found for thickness. When the thickness was 0.5mm, only 3.1% of the stimuli were perceived as surfaces; on the other hand, when the thickness was 19.5 mm, 87.9% of the stimuli were perceived to be surfaces with an evident trend shown in Table 1. It is interesting to note that, when the thickness was 3.1 mm, the choices between line and surface were about 50:50 making the stimulus visually ambiguous.

The percentage of choices which considered the stimuli as a surface was higher for straight lines (47.5% vs. 42.6%).

As far as the combination of colour (black/white) and background is concerned, when the colour was white a greater percentage of choices for surface was found (47.2% vs. 42.8%); furthermore, a grey background slightly increases the percentage of choices both when the colour was black and when the colour was white.

The evaluation of the stimuli as surfaces is stronger (in decreasing order) in case of white on grey, white on black, black on grey, and black on white.

As far as the orientation is concerned, the percentage of choices for surface was nearly the same for all the orientations.

**Figure 3:**
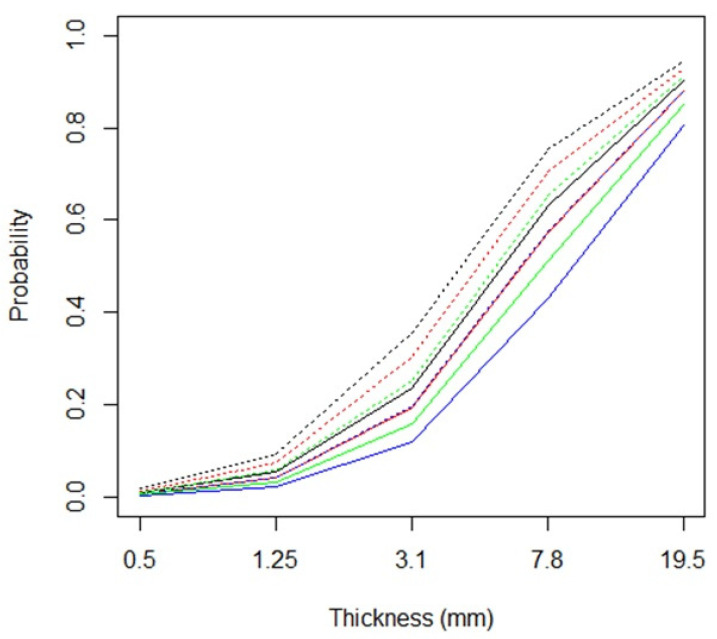
Probability of considering a stimulus as a surface according to thickness (on the abscissa, logarithmic scale), type and colour/background combination. Red lines identify white stimuli with a black background; black lines identify white stimuli with a grey background; blue lines identify black stimuli with a white background; green lines identify black stimuli with a grey background. Dotted lines identify a straight stimulus; continuous lines identify a curved stimulus.

When all these variables were considered together in a multivariate logistic regression including the random effects of participants, only orientation was not significantly associated with the response variable. The results of these analyses are shown in Figure 3. The most evident effect is the higher probability of seeing a surface when the thickness increases.

Holding thickness constant, a straight stimulus is more likely to be perceived as a surface; in the figure, when considering two lines of the same colour, the dotted one (which identifies a straight stimulus) is always above the corresponding continuous line (which identifies a curved stimulus).

The four different colours represented in Figure 3 identify the four combinations of colours (black/white) and backgrounds (black/grey/white). Red lines identify white stimuli with a black background; black lines identify white stimuli with a grey background; blue lines identify black stimuli with a white background; green lines identify black stimuli with a grey background.

For the same thickness, black ‘curved’ stimuli with a white background are those with the lowest probability of being perceived as a surface, while white ‘straight’ stimuli with a grey background are those with the highest probability of being perceived as a surface.

It may be interesting to note that in two cases the estimated probabilities are nearly the same, so that the two corresponding trends are practically superimposed. They identify black ‘straight’ stimuli on a white background and white ‘curved’ stimuli on a black background. In two other cases, the trends overlap considerably. These are black ‘straight’ stimuli on a grey background and white ‘curved’ stimuli on a grey background.

#### 2.4.2. Experiment 2

**Stimuli**

The line segments, one straight and one curved line with four different orientations and five levels of thicknesses, were presented in four chromatic colours (light blue, dark blue, yellow, and brown) on three achromatic backgrounds (white, black, and grey) for a total of 480 combinations (presented in random order).

**Task and Procedure**

The participants were given the following instructions:

The experiment consists of the subjective evaluation of line segments that appear on the screen. The line segments will be randomly presented in four colours on black, white, and grey backgrounds respectively. The participant must express her subjective perception whether the line segments are lines or surfaces. There are no wrong answers, the evaluation is subjective. However, please pay close attention to the task. Participants have as much time as desired to perform the assessment task, but must try not to respond based on past experience.

Since the total number of line segments (480) was too high for each subject, the 26 participants were divided in three different groups. The first group (*n* = 9) evaluated the line segments on a white background. The second group (*n* = 8) evaluated the line segments randomly on a grey background. The third group (*n* = 9) evaluated the line segments randomly on a black background. Each subject saw 160 line segments randomly appearing on the screen. The participants were asked to evaluate whether the line segments were lines or surfaces. The 160 line segments were given by the combination of 5 thicknesses, 2 types, 4 orientations and 4 colours.

**Results**

Table 2 show the number of choices (line/surface) made by the 26 subjects according to the variables considered. When the random effect of subjects was taken into account, a significant effect was found only for thickness.

The most striking effect was found, as expected, for thickness. When the thickness was 0.5 mm, only 2.9% of the choices considered the stimuli as a surface; on the other hand, when the thickness was 19.5 mm, 90.5% of the choices considered the stimuli as a surface with an evident trend showed in Table 2.

The percentage of choices that considered the stimuli as a surface was similar for straight and curved lines (39.6% vs. 41.8%), which differs from the results of Experiment 1 with achromatics.

The percentage of choices which considered the stimuli as a surface was similar for the four colours considered (about 41%). When the background colour was white a numerically lower percentage of choices for surface was found (33.1%), while when the background colour was grey the percentage of choices for surface was above 50%. A black background showed an intermediate value (39.7%). However, when a repeated measures analysis was made which accounted for the subject effect, these differences were not significant. Very similar results were found when the effect of background was evaluated separately for the four colours considered.

As far as the orientation is concerned, the percentage of choices for surface was nearly the same for all the orientations.

When all these variables were considered together in a multivariate logistic regression including the random effects of participants, in addiction to thickness, which remained significantly associated with the response variable, the effect of type also became significant. Even if the most important effect is the higher probability of seeing a surface when the thickness increases, holding thickness constant, the odds of considering a surface as a straight stimulus is about 1.3 times that of a curved stimulus.

#### 2.4.3. Experiment 3: OD with Achromatic

**Stimuli**

Experiment 3 considered the same line segments as Experiment 1.

**Task and Procedure**

In this case, 28 participants were asked to evaluate the line segments according to a series of 15 pairs of contraries.

The list of contraries to be evaluated with achromatic line segments was the following:

Pesante/leggera (heavy/lightweight), forte/debole (strong/weak), fredda/calda (cold/warm), femminile/maschile (feminine/masculine), piatta/arrotondata (flat/rounded), dinamica/statica (dynamic/static), accelerante/decelerante (accelerant/decelerant), centrifuga/centripeta (centrifugal/centripetal), acida/dolce (sour/sweet), smussata/tagliente (blunt/sharp), vincolata/libera (bound/free), ascendente/discendente (ascending/descending), attiva/passiva (active/passive), decrescente/crescente (decreasing/increasing), geometrica/organica (geometric/organic).

In particular the pairs accelerant/decelerant, ascending/descending, and increasing/decreasing have been chosen considering Brentano’s theory of perceptual continua [4]; the pairs dynamic/static and centrifugal/centripetal, considering Kandinsky [24] and Arnheim [7,20] on visual forces.

The participants were given the following instructions:

The experiment consists of the subjective evaluation of line segments that appear on the screen, according to a series of pairs of contraries. The line segments will be randomly presented in black and white on white, black, and grey backgrounds. There are no wrong answers, the evaluation is subjective. However, please pay close attention to the task. Participants have as much time as desired to perform the assessment task, but must try not to respond based on past experience.

**Statistical Methods**

For each pair of adjectives, the frequency distribution of the modalities of each of the considered variables was calculated. The chi-square test for the goodness of fit to a uniform distribution (i.e., the expected distribution if the choices within each pair were at random) was calculated. Since a total of 60 chi square test were performed (i.e., 15 pairs of adjectives by 4 variables), the Bonferroni correction was applied. A test was considered significant at the 1% level when the test for each individual hypothesis was significant at the 0.017% (i.e., 0.01/60). A test was considered significant at the 0.1% level when the test for each individual hypothesis was significant at the 0.0017% (i.e., 0.001/60). As a measure of size effect, the odds ratio was used. For variables having more than two categories, the highest among the possible odds ratios was selected.

**Results**

Table 3 shows the results of the chi square tests for a random choice within each pair of adjectives together with the odds ratios associated, according to the variables considered.

For variables having more than two categories, the highest among the possible odds ratios was selected. In the Supplementary Materials, the odds corresponding to each of the levels of the variables considered are reported, making it possible to calculate all the odds ratios.

When thickness was considered, a random choice can be ruled out for the following pairs: heavy/lightweight, strong/weak, cold/warm, feminine/masculine, dynamic/static, centrifugal/centripetal, blunt/sharp, bound/free, active/passive. However, when considering the pairs dynamic/static, centrifugal/centripetal, active/passive, the odds of choosing the first member were greater than 1 for all 5 thickness levels considered. Therefore, even if a random choice could be excluded, these pairs do not seem to characterise the variable thickness. The highest odds ratios were found for the pairs heavy/lightweight and strong/weak. Much lower values were found for the pairs cold/warm (with thicker lines considered warm and less thick lines considered cold), feminine/masculine (less thick lines were considered feminine, while thicker lines were considered masculine), blunt/sharp (less thick lines were considered sharp, thicker lines were considered blunt), bound/free (less thick lines were considered free, thicker lines were considered bound).

When type was considered, a random choice can be ruled out for all the pairs considered but accelerant/decelerant, ascending/descending, decreasing/increasing. However, when considering the pair centrifugal/centripetal, the odds of choosing the adjective “centrifugal” was greater than 1 for both the levels considered. Therefore, this pair does not seem to characterise the variable type. The highest odds ratios were found for the pairs (the first adjective of each pair refers to the straight stimulus and the second to the curved stimulus): geometrical/organic, flat/rounded, static/dynamic, sharp/blunt, bound/free, sour/sweet, masculine/feminine, cold/warm all with odds ratios greater than 9. A lower effect size was found for the pairs passive/active, strong/weak, heavy/lightweight. When the combination of colour (black/white) and background was evaluated, a random choice can be ruled out for the following pairs: active/passive, dynamic/static, centrifugal/centripetal, cold/warm. However, for all these pairs the odds of choosing the first member were greater than 1 for all 4 levels considered. Therefore, even if a random choice could be excluded, these pairs do not seem to characterise the combination of colour and background.

When orientation was considered, a random choice can be ruled out for the following pairs: ascending/descending, decreasing/increasing, accelerant/decelerant, centrifugal/centripetal, active/passive, dynamic/static, cold/warm, strong/weak. However, when considering the last four pairs, the odds of choosing the first member were greater than 1 for all 4 levels considered. Therefore, even if a random choice could be excluded, these pairs do not seem to characterise the variable orientation. The highest odds ratios were found for the pairs: ascending/descending, decreasing/increasing, accelerant/decelerant, centrifugal/centripetal; the significance of the result is mainly due to the diagonal stimuli.

#### 2.4.4. Experiment 4: OD with Chromatic Stimuli

**Stimuli**

The same line segments of Experiment 2 were considered.

**Task and Procedure**

In this case, 25 participants were asked to evaluate the line segments according to a series of 15 pairs of contraries.

The list of contraries to be evaluated with chromatic stimuli was the following:

Pesante/leggera (heavy/lightweight), forte/debole (strong/weak), fredda/calda (cold/warm), femminile/maschile (feminine /masculine), piatta/arrotondata (flat/rounded), dinamica/statica (dynamic/static), accelerante/decelerante (accelerant/decelerant), centrifuga/centripeta (centrifugal/centripetal), acida/dolce (sour/sweet), silenziosa/sonora (silent/sonorous), consonante/dissonante (consonant/dissonant), morbida/ruvida (fluffy/rough), frigida/sensuale (frigid/sensual), dura/molle (hard/soft), agitata/calma (agitated/calm).

The first 9 pairs were also employed in Experiment 3.

The participants were given the following instructions:

The experiment consists of the subjective evaluation of line segments that appear on the screen, according to a series of pairs of contraries. The line segments will be randomly presented in four colours on black and white backgrounds respectively. There are no wrong answers, the evaluation is subjective. However, please pay close attention to the task. Participants have as much time as desired to perform the assessment task, but must try not to respond based on past experience.

**Statistical Methods**

For each pair of adjectives, the frequency distribution of the modalities of each of the considered variables was calculated. The chi-square test for the goodness of fit to a uniform distribution (i.e., the expected distribution if the choices within each pair were at random) was calculated. Since a total of 75 chi square test were performed (i.e., 15 pairs of adjectives by 5 variables), the Bonferroni correction was applied. A test was considered significant at the 1% level when the test for each individual hypothesis was significant at the 0.013% (i.e., 0.01/75). A test was considered significant at the 0.1% level when the test for each individual hypothesis was significant at the 0.0013% (i.e., 0.001/75). As a measure of size effect, the odds ratio was used. For variables having more than two categories, the highest among the possible odds ratios was selected.

**Results**

Table 4 shows the results of the chi square tests for a random choice within each pair of adjectives together with the odds ratios associated, according to the variables considered.

For variables having more than two categories, the highest among the possible odds ratios was selected. In the Supplementary Materials, the odds corresponding to each of the levels of the variables considered are reported, making it possible to calculate all the odds ratios.

When thickness was considered, a random choice can be ruled out for the following pairs: heavy/lightweight, strong/weak, silent/sonorous, cold/warm. However, when considering the pair cold/warm, the odds of choosing cold were greater than 1 for all 5 thickness levels considered. Therefore, even if a random choice could be excluded, this pair does not seem to characterise the variable thickness. The highest odds ratios were found for the pairs heavy/lightweight, strong/weak, sonorous/silent; the second adjective characterises thinner thicknesses.

When type was considered, a random choice can be ruled out for all the pairs considered but consonant/dissonant, accelerant/decelerant, centrifugal/centripetal. However, when considering the pairs heavy/lightweight and cold/warm, the odds of choosing the adjectives cold and lightweight were greater than 1 for both the levels considered. Therefore, these pairs do not seem to characterise the variable type. The highest odds ratios were found for the pairs (the first adjective of each pair refers to the straight stimulus and the second to the curved stimulus): flat/rounded, hard/soft, static/dynamic, rough/fluffy, frigid/sensual, masculine/feminine, sour/sweet all with odds ratios greater than 6. Less evidence was found for the pairs silent/sonorous, calm/agitated, strong/weak.

When colour was considered, a random choice can be ruled out for the pairs cold/warm, silent/sonorous, agitated/calm, heavy/lightweight, frigid/sensual. However, when considering the pair heavy/lightweight, the odds of choosing the adjective lightweight were greater than 1 for 4 levels considered. Therefore, this pair does not seem to characterise the variable colour. An important effect size was found only for the pair cold/warm. Yellow lines were considered warmer, while light blue and blue were considered the most cold; brown lines were considered colder than warmer. Less evidence was found for the remaining three pairs: yellow lines were considered sonorous, calm and sensual; light blue as well as blue lines were considered agitated, frigid and silent; brown lines were considered silent, frigid and calm.

On the other hand, when background was considered, a random choice can be ruled out for the pairs cold/warm and heavy/lightweight. However, the odds of choosing the adjectives cold and lightweight were greater than 1 for all 3 levels considered. Therefore, these pairs do not seem to characterise the variable background.

When orientation was considered, a random choice can be ruled out for the following pairs: accelerant/decelerant, cold/warm, lightweight/heavy. However, when considering the last two pairs, the odds of choosing the first member were greater than 1 for all 4 levels considered. Therefore, even if a random choice could be excluded, these two pairs do not seem to characterise the variable orientation. The highest odds ratio for the pair accelerant/decelerant was 6.2; the relevance of the result is mainly due to the diagonal stimuli.

## 3. Discussion

### 3.1. Comparison between the Results of Experiments 1 and 2

In this study, we evaluated five variables possibly responsible for a visual appearance to be perceived as a line or as a surface. These variables were type, thickness, orientation, colour (chromatic and achromatic) and background, to verify their respective weight in shape categorisation.

We conducted four experiments, two based on the subjective evaluation of the line segments (Experiment 1 and 2), and two with the Osgood semantic differential (Experiment 3 and 4).

Comparing the results of the first two experiments on subjective evaluations of the line segments, we obtained the following results.

Both with achromatic and chromatic line segments, as expected, we found that thickness dominates over all the other variables; furthermore, the result sheds light on other characteristics. It is interesting to note that, with achromatic line segments, when the thickness was 3.1 mm, the line segment appeared visually ambiguous, and consequently it is possible to argue for a perceptual boundary between the two categories among the line segments that we considered. On the other hand, with the same thickness and chromatic line segments, 1/3 of the subjects evaluated the stimulus as a surface, while 2/3 evaluated the same stimulus as line. In this case, the boundary between the two categories, would have been between 3 and 8mm. Therefore, it appears that the hues used have reduced the probability of perceiving the stimuli as surfaces.

It is worth noting that when the thicknesses were extreme, the subjects’ answers were not unanimous.

As for the variable type, with achromatic stimuli, our analysis showed the tendency for straight lines to be categorised as surfaces, and vice versa for curved lines, while with chromatic stimuli the variable type was not influential. On the other hand, in the multivariate analysis, this variable was significantly associated with the response both with achromatic and chromatic stimuli.

In the case of achromatic stimuli obviously it is not possible to differentiate the influence of colour from the influence of the background. Generally, white stimuli were more frequently categorised as surfaces; grey backgrounds appeared to play a significant role in the choice. The results showed the incidence of factors such as colour assimilation, colour expansion/contraction connotative property, and the contrast between figure and ground. Specifically, stimuli categorised as surfaces were (in decreasing order): white stimuli on grey background (the effect is probably due to the expansion and the assimilation effect of white, and to the figure/ground contrast); white stimuli on black (the effect is probably due to the expansion effect of white and to the figure/ground contrast); black stimuli on grey background (the effect is probably due to the contraction effect of black and to the figure/ground contrast); black stimuli on white (the effect is probably due to the contraction effect of black and to the figure/ground contrast).

As for the contraction effect of the black stimuli, this seems to be accentuated by a lighter (white) background and softened by a darker (grey) background. Surprisingly, the expansion effect of white is dampened by a darker (black) background and slightly accentuated by a lighter (grey) background. One possible explanation of this result is that the effect is due to the colour assimilation of figure and background, or that it occurs when the margins between figure and background are less sharp (white on grey and black on grey).

With chromatic stimuli, the percentage of choices which perceived the stimuli as a ‘surface’ was similar for the four hues considered. Very similar results were found when the effect of background was evaluated separately for the four hues considered.

For both the achromatic and chromatic stimuli, the orientation was not found to be significant. A different grouping of the orientation variable was also considered. The converging and diverging edges of the stimuli can be perspective cues for perceived depth. However, taking the orientations 3, 5 and 7 together as one group and orientations 4, 6 and 8 as another (see Figure 1), no significant difference in the proportion of line/surface choice was found.

Briefly, for what concerns the first two Experiments (1 and 2) conducted with the methods of subjective evaluations by the participants, when all the variables were considered together in a multivariate logistic regression including the random effects of participants, thickness and type were significantly associated with the response variable, although the most important effect is the higher probability of seeing a surface when the thickness increases. Surprisingly, the variable colour was significant only for achromatic stimuli, where the results showed the predominance of the expansion-contraction and contrast effects.

### 3.2. Comparison between the Results of Experiments 3 and 4

Comparing the results of the other two Experiments (3 and 4) conducted with the Osgood semantic differential, we obtained the following results.

When achromatic stimuli were evaluated according to the list of contraries (see Experiment 3), for what concerns the variable thickness, a strong effect was found for the pairs of adjectives heavy/lightweight and strong/weak, which was expected. Much lower values were found for the pairs cold/warm, feminine/masculine, blunt/sharp, bound/free. These results offer new information about the warmth connotative property of lines, which have been considered warm/cold only in relation to their horizontal/vertical orientation [24] (ch. 2); and, to our knowledge, also in relation to their expressive property feminine/masculine.

When chromatic stimuli were evaluated according to the list of contraries (see Experiment 4), for what concerns the variable thickness, the strong effect of the two pairs heavy/lightweight and strong/weak was confirmed; in addition, also the pair silent/sonorous showed a high effect size; this is apparently a confirmation of Kandinsky’s idea of the acoustic cross-modal property of colour.

When achromatic stimuli were evaluated, for the variable type a strong effect was found for the pairs (the first adjective of each pair refers to the straight stimulus and the second to the curved stimulus): geometrical/organic, flat/rounded, static/dynamic, sharp/blunt, bound/free, sour/sweet, masculine/feminine, cold/warm, passive/active.

When chromatic stimuli were evaluated, for the variable type a strong effect was found for the pairs: flat/rounded, hard/soft, static/dynamic, rough/fluffy, frigid/sensual, masculine/feminine, sour/sweet. A much lower effect also was found for the pairs calm/agitated and silent/sonorous.

With chromatic stimuli, orientation had an important effect only for the pair accelerant/decelerant (which was expected). A similar effect was found when achromatic stimuli were considered; in addition, the pairs ascending/descending and decreasing/increasing showed a stronger effect size.

When chromatic stimuli were considered in Experiment 4 for the variable colour, a strong effect was found for the pair cold/warm. This was expected, because of the strength of the dimension warmth in colour appearances [44,45]. A lower effect was found for the pairs agitated/calm, silent/sonorous, frigid/sensual, while for background, no adjective showed an important effect size. A similar result was found also with achromatic stimuli.

Some of our expectations were confirmed, for example the importance of the two pairs heavy/lightweight and strong/weak for the variable thickness; the importance of pairs of adjectives characterising perceived velocity such as ascending/descending, accelerant/decelerant, decreasing/increasing for the variable orientation; and the importance of pair of adjectives characterising warmth for the variable colour. It is worth noticing the importance of cross-modal (sour/sweet, fluffy/rough) and expressive (feminine/masculine, active/passive, frigid/sensual) pairs of adjectives for the variable type (shape), with achromatic and/or chromatic stimuli; and the frequent overlapping of the pairs feminine/masculine, flat/rounded, dynamic/static, sour/sweet in both stimuli.

More generally, as occurred with the subjective evaluations of the stimuli (Experiment 1 and 2), in the Experiments conducted with the Osgood semantic differential (Experiment 3 and 4), the most relevant results occurred with the achromatic stimuli.

### 3.3. General Results

To summarise, as to the difference between the results of the experiments conducted with the method of subjective evaluations, and the results of the experiments conducted with the method of the Osgood semantic differential, we obtained similar results: higher consistency of the results with achromatic line segments than with chromatic ones; higher importance of the variables thickness and type (for both achromatic and chromatic line segments); general lower significance of the variable colour (although with a difference between achromatic and chromatic line segments, confirmed by the relevance of the pair cold/warm only); and general insignificance of the variable orientation. Interestingly, for the variable type, by the Osgood semantic differential straight line segments were categorised as geometrical, hard, rough, sharp, bound, frigid, passive, flat, static, sour, masculine and cold; the last five adjectives were common to achromatic and chromatic line segments. Curved lines were characterised by the second term of the pairs listed above.

The categorisation that opposes straight lines to curved lines matches the opposition of the two types of line segments; besides the consistency of the categorisation, which opposed straight to curved line segments, as expected based on studies in the arts [25,44] (ch. 2), the results obtained with Osgood semantic differential offered interesting semantic information as to the cross-modal and expressive meaning naturally conveyed by visual shapes (types).

### 3.4. Conclusions

Interestingly, and contrary to what we expected, the colour variable was not particularly relevant in influencing the categorisation, except for the achromatic line segments. The same goes for the colour of the variable background, which was important only for achromatic line segments and with a strong relevance of grey. The reasons why colour does not have a significant effect might be due to the fact that lines do not stand out enough from each other for chromatic and dimensional aspects. Firstly, lines easily cause assimilation effects [46], which results in a minor distinction from the background. Secondly, the isoluminance of colours, which is a usual condition in colour science, is not phenomenologically appropriate. The missing chromatic effect we predicted seems to depend on the fact that perceptively the chosen colours are not sufficiently distinguishable from each other in the context in which they are presented because of their isoluminance. Possibly, we would have obtained a better perceptual discrimination if we had chosen colours having a lightness congruent with the natural lightness of their hue.

As to the question posed at the beginning of our study, i.e., the possibility to identify a boundary between visual lines and visual surfaces, given the five dimensions of thickness (0.5 to 19.5 mm) that we considered, we may conclude that, as to the achromatic line segments, the boundary was estimated at about the third value of the thickness’ choices (3.1 mm).

In the light of the relevance of the variable thickness showed by our results, further studies could diversify and add more degree of thickness of the line segments, giving particular attention to the length of lines. As mentioned, in our experiments both types of line segments were chosen to cover broadly the same amount of space between the two extremities because according to the type they may appear of different length and consequently influence the evaluation of some variables. One might intentionally draw line segments of different length instead, both straight and curved ones.

Most interestingly, one might present the line segments not on Euclidean flat dimensional surfaces but on closed and curved surfaces; or present the line segments at different perceived depth.

Another interesting study would be to explore the cross-modal relationship between our (or similar) lines with sound.

Considering the not significant results for the chromatic colours, further studies might use different hues, considering their natural lightness. This choice might also give clearer results if line segments were chosen based on the connotative properties of colour (warmth, and expansion/contraction effect of hues).

Finally, our study has been conducted in the framework and with the methods of experimental phenomenology. We did not address the issue of the neurophysiological substrate of the subjective discrimination between visual lines and surfaces. For the time being, and to our knowledge, there are no comparable studies in the field of neuroscience, nor can our results be compared with those obtained in studies concerning contours, junctions or edges in the field of visual object recognition. It would be desirable that studies such as ours could be developed in the future in the neuroscientific field, to explore the correlations. However, from a phenomenological viewpoint, besides the methods of subjective evaluations, the best information on elements of visual space (point, lines, surfaces) are still provided by art. In our case, the types of lines resembled Kandinsky’s and for this reason, as mentioned, they required the expertise of a professional graphic designer, able to keep a natural variation in thickness and their cross-modal characteristics. The results of our study, in fact, can be useful for designers.

In the light of this assumption the results, although preliminary, shed light on the characteristics of an element of visual space that can contribute to conceiving a proper geometry of seeing. Such a geometry should consider perceptual, expressive and crossmodal dimensions of its elements. We are perfectly aware of the challenge and may only hope to have start showing the potentiality of the viewpoint. It is certainly premature to consider our results on the study of visual lines as definitive, but it can fairly be considered a first step to understand the nature of at least one of the elements of a phenomenological geometry of seeing.

Last but not least, our experiments are based on the simulation of lines as perceptual appearances. A more ambitious goal would be to conduct experiments in open space focusing on visual lines as they are seen in natural environments.

## Figures and Tables

**Table 1:** Subjects‘ choices for the independent variables considered in Experiment 1 evaluated employing a logistic model for repeated measures, where subjects are considered a random effect and the dependent variable is the probability of considering the stimulus as a surface.

Variable	Line	Surface	% Surf.	LRT	*p*
Thickness (mm)				984	<0.001
0.5	930	30	3.1		
1.25	799	161	16.8		
3.1	511	449	46.8		
7.8	283	677	70.5		
19.5	116	844	87.9		
Type				13.76	<0.001
curved	1378	1022	42.6		
straight	1261	1139	47.5		
Orientation				0.24	0.97
horizontal	657	543	45.3		
vertical	656	544	45.3		
harmonic diagonal	660	540	45.0		
disharmonious diagonal	666	534	44.5		
Colour/Background				14.84	0.002
white on grey	621	579	48.3		
white on black	645	555	46.3		
black on grey	670	530	44.2		
black on white	703	497	41.4		

Line: number of times ‘line’ has been chosen. Surface: number of times ‘surface’ has been chosen. %Surface: percentage of times ‘surface’ has been chosen. LRT: Likelihood Ratio Test for independence in a logistic model. For repeated measures. *p*: *p*-value of the Likelihood Ratio Test.

**Table 2:** Subjects‘ choices for the independent variables considered in Experiment 2 evaluated employing a logistic model for repeated measures, where subjects are considered a random effect and the dependent variable is the probability of considering the stimulus as a surface.

Variable	Line	Surface	% Surf.	LRT	*p*
Thickness (mm)				888	<0.001
0.5	808	24	2.9		
1.25	710	122	14.7		
3.1	555	277	33.3		
7.8	315	517	62.1		
19.5	79	753	90.5		
Type				2.62	0.11
curved	1257	823	39.6		
straight	1210	870	41.8		
Orientation				0.69	0.88
horizontal	611	429	41.3		
vertical	620	420	40.4		
harmonic diagonal	611	429	41.3		
disharmonious diagonal	625	415	39.9		
Colour				0.91	0.82
light blue	605	435	41.8		
dark blue	623	417	40.1		
yellow	620	420	40.4		
brown	619	421	40.5		
Background				3.38	0.18
white	964	476	33.1		
grey	635	645	50.4		
black	868	572	39.7		

Line: number of times ‘line’ has been chosen. Surface: number of times ‘surface’ has been chosen. %Surface: percentage of times ‘surface’ has been chosen. LRT: Likelihood Ratio Test for independence in a logistic model. For repeated measures. *p*: *p*-value of the Likelihood Ratio Test.

**Table 3:** Results of the chi square tests for a uniform choice and odds ratios for each pair of adjectives (Experiment 3).

	Thickness			Type			Colour/Background			Orientation		
Pairs of Adjectives	chi^2^		OR	chi^2^		OR	chi^2^		OR	chi^2^		OR
heavy/lightweight	538.89	**	522.82	30.36	**	1.66	14.09		1.17	14.47		1.22
strong/weak	362.09	**	39.56	48.89	**	1.98	22.04		1.36	25.15	*	1.59
cold/warm	67.68	**	3.16	289.43	**	9.03	26.94	*	1.23	25.62	*	1.10
feminine/masculine	46.52	**	2.92	306.61	**	10.05	14.93		1.30	17.71		1.45
flat/rounded	9.07		1.57	764.03	**	114.47	1.86		1.04	5.36		1.35
dynamic/static	42.18	**	1.39	481.14	**	25.30	40.66	**	1.25	41.43	**	1.33
accelerant/decelerant	2.23		1.28	1.21		1.13	3.81		1.37	129.01	**	7.62
centrifugal/centripetal	35.32	**	1.16	47.16	**	1.56	36.60	**	1.27	90.17	**	3.17
sour/sweet	0.68		1.13	432.51	**	18.34	2.17		1.26	1.43		1.21
blunt/sharp	28.02	*	2.58	486.32	**	23.66	0.53		1.11	5.45		1.41
bound/free	37.63	**	2.27	473.58	**	22.92	14.96		1.24	20.01		1.52
ascending/descending	10.27		1.38	5.72		1.07	14.27		1.50	250.84	**	21.43
active/passive	44.11	**	1.42	163.69	**	4.24	42.11	**	1.23	55.36	**	1.86
decreasing/increasing	0.86		1.15	0.76		1.11	9.66		1.68	227.20	**	18.64
geometric/organic	1.34		1.09	848.06	**	217.44	1.27		1.07	4.73		1.32

chi^2^: chi square test for a uniform choice between the adjectives of a pair. * *p* < 0.01 (employing the Bonferroni correction for 60 tests). ** *p* < 0.001 (employing the Bonferroni correction for 60 tests). OR: ratio between the maximum and the minimum odds of choosing the first member of a pair. The direction of the association can be retrieved in the text and in the Supplementary Materials.

**Table 4:** Results of the chi square tests for a uniform choice and odds ratios for each pair of adjectives (Experiment 4).

	Thickness			Type			Colour			Background			Orientation		
Pairs of Adjectives	chi^2^		OR	chi^2^		OR	chi^2^		OR	chi^2^		OR	chi^2^		OR
heavy/lightweight	462.74	**	248.83	49.64	**	1.30	62.00	**	2.13	52.60	**	1.53	46.17	**	1.11
strong/weak	234.96	**	27.15	44.96	**	2.37	12.99		1.85	1.85		1.20	2.61		1.31
cold/warm	80.62	**	2.27	88.23	**	1.95	339.76	**	29.18	77.73	**	1.75	67.35	**	1.35
feminine/masculine	13.62		1.69	218.60	**	7.57	7.63		1.50	7.18		1.42	5.13		1.35
flat/rounded	6.44		1.15	671.63	**	117.24	6.50		1.16	5.97		1.06	7.41		1.24
dynamic/static	12.84		1.50	543.57	**	46.69	10.27		1.32	10.07		1.25	13.09		1.43
accelerant/decelerant	2.18		1.25	4.04		1.26	3.38		1.29	1.95		1.20	109.18	**	6.17
centrifugal/centripetal	9.42		1.69	20.07	*	1.72	4.37		1.25	10.86		1.51	11.53		1.54
sour/sweet	7.40		1.59	193.86	**	6.62	13.92		1.91	0.87		1.12	3.26		1.32
silent/sonorous	102.68	**	7.22	69.15	**	2.66	85.79	**	4.05	18.34		1.39	17.16		1.38
consonant/dissonant	8.92		1.59	2.32		1.14	6.88		1.49	3.70		1.26	10.95		1.64
fluffy/rough	22.74		1.51	439.40	**	25.62	22.48		1.41	18.86		1.22	21.41		1.36
frigid/sensual	23.18		1.23	378.92	**	18.05	48.91	**	2.52	22.97	*	1.23	26.98	*	1.52
hard/soft	3.10		1.15	652.14	**	92.76	4.46		1.25	6.45		1.37	7.94		1.37
agitated/calm	7.48		1.62	41.26	**	2.70	78.87	**	6.27	6.26		1.36	4.43		1.29

chi^2^: chi square test for a uniform choice between the adjectives of a pair. * *p* < 0.01 (employing the Bonferroni correction for 75 tests). ** *p* < 0.001 (employing the Bonferroni correction for 75 tests). OR: ratio between the maximum and the minimum odds of choosing the first member of a pair. The direction of the association can be retrieved in the text and in the Supplementary Materials.

## Data Availability

The data presented in this study are available on request from the corresponding author.

## Supplementary Materials

The following are available online at https://www.mdpi.com/article/10.3390/brainsci11121585/s1, Table S1: Results of the analysis on the Osgood differential semantic (Experiment 3). Table S2: Results of the analysis on the Osgood differential semantic (Experiment 4). Achromatic_Stimuli: Folder containing all the 320 achromatic stimuli. Chromatic_Stimuli: Folder containing all the 960 chromatic stimuli.
