# Supplementary material for: The Perceptual Organisation of Visual Elements: Lines
Source: Brain Sci. 2021 Nov 30;11(12):1585. doi: 10.3390/brainsci11121585 (PMC8699471; doi:10.3390/brainsci11121585)
Supplement: Supplementary file 1 [file brainsci-11-01585-s001.zip › supplementary material - Experiment 3.pdf]

Results of the analysis on the Osgood differential semantic (Experiment 3).

chi^2: chi square tests for a uniform choice between the two adjectives of each pair.

OR: ratio between the maximun and the minimum odds of choosing the first member of a pair.

The last five columns report the odds of choosing the first member of a pair for each thickness value.

			Thickness (mm)				
	chi^2	OR	0.50	1.25	3.10	7.80	19.50
heavy/lightweight	538.89	522.82	0.037	0.161	0.672	2.394	19.364
strong/weak	362.09	39.56	0.273	0.383	1.605	3.870	10.789
cold/warm	67.68	3.16	2.500	1.667	1.517	0.965	0.792
feminine/masculine	46.52	2.92	2.111	1.489	1.286	1.018	0.723
active/passive	44.11	1.42	1.196	1.699	1.635	1.435	1.435
dynamic/static	42.18	1.39	1.383	1.667	1.517	1.575	1.196
bound/free	37.63	2.27	0.566	0.635	0.723	0.982	1.286
centrifugal/centripetal	35.32	1.16	1.333	1.409	1.517	1.545	1.333
blunt/sharp	28.02	2.58	0.577	0.898	1.196	1.133	1.489
ascending/descending	10.27	1.38	0.764	0.723	0.915	1.000	0.982
flat/rounded	9.07	1.57	0.948	0.867	1.093	1.218	1.358
accelerant/decelerant	2.23	1.28	0.898	1.000	1.000	1.093	1.154
geometric/organic	1.34	1.09	1.074	1.000	1.055	1.093	1.093
decreasing/increasing	0.86	1.15	1.055	1.036	1.055	0.915	1.018
sour/sweet	0.68	1.13	1.000	0.948	1.036	1.074	0.948

Results of the analysis on the Osgood differential semantic (Experiment 3).
chi^2: chi square tests for a uniform choice between the two adjectives of each pair.
OR: ratio between the maximun and the minimum odds of choosing the first member of a pair.

			Type	
	chi^2	OR	curved	straight
geometric/organic	848.06	217.44	0.087	19.000
flat/rounded	764.03	114.47	0.120	13.737
blunt/sharp	486.32	23.66	4.957	0.210
dynamic/static	481.14	25.30	9.769	0.386
bound/free	473.58	22.92	0.143	3.275
sour/sweet	432.51	18.34	0.233	4.283
feminine/masculine	306.61	10.05	4.234	0.421
cold/warm	289.43	9.03	0.497	4.490
active/passive	163.69	4.24	3.179	0.750
strong/weak	48.89	1.98	0.924	1.828
centrifugal/centripetal	47.16	1.56	1.786	1.146
heavy/lightweight	30.36	1.66	0.623	1.036
ascending/descending	5.72	1.07	0.898	0.842
accelerant/decelerant	1.21	1.13	1.090	0.965
decreasing/increasing	0.76	1.11	0.965	1.066

Results of the analysis on the Osgood differential semantic (Experiment 3).

chi^2: chi square tests for a uniform choice between the two adjectives of each pair.

OR: ratio between the maximun and the minimum odds of choosing the first member of a pair.

The last four columns report the odds of choosing the first member of a pair for the four categories of the variable colour/background.

			Colour/Background			
	chi^2	OR	White on Grey	White on Black	Black on White	Black on Grey
active/passive	42.11	1.23	1.353	1.353	1.523	1.667
dynamic/static	40.66	1.25	1.373	1.353	1.435	1.692
centrifugal/centripetal	36.60	1.27	1.258	1.353	1.593	1.523
cold/warm	26.94	1.23	1.456	1.353	1.435	1.188
strong/weak	22.04	1.36	1.154	1.171	1.569	1.314
bound/free	14.96	1.24	0.806	0.905	0.783	0.728
feminine/masculine	14.93	1.30	1.276	1.188	1.090	1.414
ascending/descending	14.27	1.50	1.029	0.697	1.044	0.761
heavy/lightweight	14.09	1.17	0.739	0.867	0.854	0.772
decreasing/increasing	9.66	1.68	0.761	1.276	1.014	1.074
accelerant/decelerant	3.81	1.37	1.044	0.854	1.171	1.059
sour/sweet	2.17	1.26	1.059	0.867	1.090	1.000
flat/rounded	1.86	1.04	1.105	1.059	1.059	1.105
geometric/organic	1.27	1.07	1.090	1.090	1.014	1.059
blunt/sharp	0.53	1.11	1.074	0.972	1.029	0.972

Results of the analysis on the Osgood differential semantic (Experiment 3).

chi^2: chi square tests for a uniform choice between the two adjectives of each pair.

OR: ratio between the maximun and the minimum odds of choosing the first member of a pair.

The last four columns report the odds of choosing the first member of a pair for the four categories of the variable orientation.

			Orientation			
	chi^2	OR	Horizontal	Vertical	Harmonic Diagonal	Disharmonious Diagonal
ascending/descending	250.84	21.43	1.119	0.669	0.186	3.982
decreasing/increasing	227.20	18.64	0.832	1.165	4.263	0.229
accelerant/decelerant	129.01	7.62	1.135	0.819	0.422	3.215
centrifugal/centripetal	90.17	3.17	2.054	1.079	0.807	2.558
active/passive	55.36	1.86	1.219	1.220	1.419	2.262
dynamic/static	41.43	1.33	1.290	1.382	1.479	1.713
cold/warm	25.62	1.10	1.407	1.278	1.326	1.404
strong/weak	25.15	1.59	1.254	1.063	1.239	1.686
bound/free	20.01	1.52	0.986	0.735	0.863	0.651
feminine/masculine	17.71	1.45	1.559	1.202	1.158	1.076
heavy/lightweight	14.47	1.22	0.786	0.782	0.911	0.745
blunt/sharp	5.45	1.41	1.058	1.259	0.899	0.890
flat/rounded	5.36	1.35	1.058	0.899	1.174	1.210
geometric/organic	4.73	1.32	0.945	0.955	1.128	1.246
sour/sweet	1.43	1.21	0.919	1.113	0.961	1.030
