# Supplementary material for: The Perceptual Organisation of Visual Elements: Lines
Source: Brain Sci. 2021 Nov 30;11(12):1585. doi: 10.3390/brainsci11121585 (PMC8699471; doi:10.3390/brainsci11121585)
Supplement: Supplementary file 1 [file brainsci-11-01585-s001.zip › supplementary material - Experiment 4.pdf]

Results of the analysis on the Osgood differential semantic (Experiment 4).

chi^2: chi square tests for a uniform choice between the two adjectives of each pair.

OR: ratio between the maximun and the minimum odds of choosing the first member of a pair.

The last five columns report the odds of choosing the first member of a pair for each thickness value.

			Thickness (mm)				
	chi^2	OR	0.50	1.25	3.10	7.80	19.50
heavy/lightweight	462.74	248.83	0.031	0.111	0.471	1.703	7.696
strong/weak	234.96	27.15	0.258	0.399	0.942	1.778	7.000
silent/sonorous	102.68	7.22	3.167	1.632	1.439	1.062	0.439
cold/warm	80.62	2.27	2.774	1.817	1.667	1.381	1.222
frigid/sensual	23.18	1.23	1.469	1.222	1.469	1.381	1.198
fluffy/rough	22.74	1.51	1.083	1.198	1.500	1.174	1.632
feminine/masculine	13.62	1.69	1.174	1.128	1.020	0.724	0.695
dynamic/static	12.84	1.50	1.198	1.174	1.353	1.381	0.923
centrifugal/centripetal	9.42	1.69	0.835	1.041	1.198	1.410	1.083
consonant/dissonant	8.92	1.59	1.041	1.273	1.353	0.942	0.852
agitated/calm	7.48	1.62	1.509	1.197	1.120	1.024	0.933
sour/sweet	7.40	1.59	1.410	1.083	0.961	0.887	0.905
flat/rounded	6.44	1.15	1.247	1.083	1.151	1.222	1.128
hard/soft	3.10	1.15	1.151	1.174	1.020	1.105	1.083
accelerant/decelerant	2.18	1.25	0.887	0.961	0.961	0.869	1.083

Results of the analysis on the Osgood differential semantic (Experiment 4).
chi^2: chi square tests for a uniform choice between the two adjectives of each pair.
OR: ratio between the maximun and the minimum odds of choosing the first member of a pair.

			Type	
	chi^2	OR	curved	straight
flat/rounded	671.63	117.24	0.149	17.519
hard/soft	652.14	92.76	0.139	12.889
dynamic/static	543.57	46.69	10.111	0.217
fluffy/rough	439.40	25.62	8.091	0.316
frigid/sensual	378.92	18.05	0.377	6.812
feminine/masculine	218.60	7.57	2.497	0.330
sour/sweet	193.86	6.62	0.404	2.676
cold/warm	88.23	1.95	1.222	2.378
silent/sonorous	69.15	2.66	0.786	2.086
heavy/lightweight	49.64	1.30	0.567	0.736
strong/weak	44.96	2.37	0.645	1.525
agitated/calm	41.26	2.70	1.498	0.554
centrifugal/centripetal	20.07	1.72	1.439	0.838
accelerant/decelerant	4.04	1.26	1.066	0.845
consonant/dissonant	2.32	1.14	1.146	1.008

Results of the analysis on the Osgood differential semantic (Experiment 4).

chi^2: chi square tests for a uniform choice between the two adjectives of each pair.

OR: ratio between the maximun and the minimum odds of choosing the first member of a pair.

The last four columns report the odds of choosing the first member of a pair for the four categories of the variable colour.

			Colour			
	chi^2	OR	Light Blue	Dark Blue	Yellow	Brown
cold/warm	339.76	29.18	9.417	4.952	0.323	1.119
silent/sonorous	85.79	4.05	1.976	1.747	0.488	1.525
agitated/calm	78.87	6.27	4.083	2.214	0.651	0.890
heavy/lightweight	62.00	2.13	0.420	0.712	0.634	0.894
frigid/sensual	48.91	2.52	2.012	1.604	0.799	1.294
fluffy/rough	22.48	1.41	1.551	1.101	1.475	1.137
sour/sweet	13.92	1.91	0.969	1.404	1.137	0.736
strong/weak	12.99	1.85	0.786	0.894	1.451	0.953
dynamic/static	10.27	1.32	1.232	1.137	1.381	1.049
feminine/masculine	7.63	1.50	1.033	0.748	1.119	0.852
consonant/dissonant	6.88	1.49	1.252	1.119	1.137	0.838
flat/rounded	6.50	1.16	1.273	1.155	1.101	1.137
hard/soft	4.46	1.25	0.953	1.174	1.119	1.193
centrifugal/centripetal	4.37	1.25	1.193	0.969	1.033	1.212
accelerant/decelerant	3.38	1.29	0.880	0.838	1.083	1.016

Results of the analysis on the Osgood differential semantic (Experiment 4).
chi^2: chi square tests for a uniform choice between the two adjectives of each pair.
OR: ratio between the maximun and the minimum odds of choosing the first member of a pair.
The last four columns report the odds of choosing the first member of a pair for the three categories of the variable background.

			Background		
	chi^2	OR	White	Grey	Black
cold/warm	77.73	1.75	2.364	1.478	1.353
heavy/lightweight	52.60	1.53	0.519	0.795	0.682
frigid/sensual	22.97	1.23	1.483	1.205	1.323
fluffy/rough	18.86	1.22	1.222	1.205	1.466
silent/sonorous	18.34	1.39	1.093	1.205	1.517
centrifugal/centripetal	10.86	1.51	0.856	1.222	1.293
dynamic/static	10.07	1.25	1.081	1.154	1.353
feminine/masculine	7.18	1.42	1.130	0.795	0.856
hard/soft	6.45	1.37	1.118	1.314	0.957
agitated/calm	6.26	1.36	1.290	1.204	0.948
flat/rounded	5.97	1.06	1.209	1.137	1.143
consonant/dissonant	3.70	1.26	1.022	0.972	1.222
accelerant/decelerant	1.95	1.20	1.022	0.854	0.957
strong/weak	1.85	1.20	1.057	1.059	0.885
sour/sweet	0.87	1.12	0.967	1.059	1.081

Results of the analysis on the Osgood differential semantic (Experiment 4).

chi^2: chi square tests for a uniform choice between the two adjectives of each pair.

OR: ratio between the maximun and the minimum odds of choosing the first member of a pair.

The last four columns report the odds of choosing the first member of a pair for the four categories of the variable orientation.

			Orientation			
	chi^2	OR	Horizontal	Vertical	Harmonic Diagonal	Disharmonious Diagonal
accelerant/decelerant	109.18	6.17	1.185	0.626	0.389	2.398
cold/warm	67.35	1.35	1.408	1.779	1.641	1.907
heavy/lightweight	46.17	1.11	0.639	0.660	0.609	0.679
frigid/sensual	26.98	1.52	1.291	1.060	1.613	1.452
fluffy/rough	21.41	1.36	1.538	1.464	1.132	1.153
silent/sonorous	17.16	1.38	1.126	1.255	1.558	1.169
dynamic/static	13.09	1.43	0.967	1.096	1.382	1.350
centrifugal/centripetal	11.53	1.54	1.360	0.882	0.913	1.293
consonant/dissonant	10.95	1.64	1.511	1.078	0.929	0.918
hard/soft	7.94	1.37	0.950	0.943	1.292	1.256
flat/rounded	7.41	1.24	1.226	1.043	1.292	1.120
feminine/masculine	5.13	1.35	1.088	0.853	1.008	0.808
agitated/calm	4.43	1.29	0.959	1.140	1.163	1.237
sour/sweet	3.26	1.32	0.858	1.134	1.025	1.120
strong/weak	2.61	1.31	1.034	0.943	1.150	0.880
